# The Association Between Short-term Exposure to Ambient Air Pollution and Patient-Level Home Blood Pressure Among Patients With Chronic Cardiovascular Diseases in a Web-Based Synchronous Telehealth Care Program: Retrospective Study

**DOI:** 10.2196/26605

**Published:** 2021-06-08

**Authors:** Ching-Chang Huang, Ying-Hsien Chen, Chi-Sheng Hung, Jen-Kuang Lee, Tse-Pin Hsu, Hui-Wen Wu, Pao-Yu Chuang, Ming-Fong Chen, Yi-Lwun Ho

**Affiliations:** 1 Telehealth Center National Taiwan University Hospital Taipei Taiwan; 2 Department of Internal Medicine National Taiwan University Hospital Taipei Taiwan; 3 Department of Nursing National Taiwan University Hospital Taipei Taiwan

**Keywords:** ambient air pollution, blood pressure, cardiovascular disease, chronic disease, climate, home blood pressure, particulate matter, pollution, remote monitoring, telehealth care, telemonitoring, weather

## Abstract

**Background:**

The association between short-term exposure to ambient air pollution and blood pressure has been inconsistent, as reported in the literature.

**Objective:**

This study aimed to investigate the relationship between short-term ambient air pollution exposure and patient-level home blood pressure (HBP).

**Methods:**

Patients with chronic cardiovascular diseases from a telehealth care program at a university-affiliated hospital were enrolled as the study population. HBP was measured by patients or their caregivers. Hourly meteorological data (including temperature, relative humidity, wind speed, and rainfall) and ambient air pollution monitoring data (including CO, NO_2_, particulate matter with a diameter of <10 µm, particulate matter with a diameter of <2.5 µm, and SO_2_) during the same time period were obtained from the Central Weather Bureau and the Environmental Protection Administration in Taiwan, respectively. A stepwise multivariate repeated generalized estimating equation model was used to assess the significant factors for predicting systolic and diastolic blood pressure (SBP and DBP).

**Results:**

A total of 253 patients and 110,715 HBP measurements were evaluated in this study. On multivariate analysis, demographic, clinical, meteorological factors, and air pollutants significantly affected the HBP (both SBP and DBP). All 5 air pollutants evaluated in this study showed a significant, nonlinear association with both home SBP and DBP. Compared with demographic and clinical factors, environmental factors (meteorological factors and air pollutants) played a minor yet significant role in the regulation of HBP.

**Conclusions:**

Short-term exposure to ambient air pollution significantly affects HBP in patients with chronic cardiovascular disease.

## Introduction

Air pollution is a great hazard to public health [1-5]. Previous studies have shown that long-term exposure to ambient air pollution increases respiratory morbidity and cardiopulmonary disease–related mortality [6,7], while short-term exposure increases cardiovascular disease–related mortality and nonfatal events [8]. The most prominent air pollutants that potentially affect human health include nitrogen oxides (NOx, including NO_2_ and NO), CO, O_3_, SO_2_, particulate matter with a diameter of <10 μm (PM_10_), and particulate matter with a diameter of <2.5 μm (PM_2.5_). Hypertension is among the most prominent risk factors contributing to cardiovascular diseases, and the potential detrimental effect of air pollution on blood pressure (BP) is considered one of the mechanisms underlying the exacerbation in cardiovascular outcomes. However, the association between short-term or long-term exposure to ambient air pollution and BP has been inconsistent in previous studies, and most of these studies were epidemiological [9-12]. In this retrospective study, we aimed to evaluate the effect of short-term exposure to ambient air pollution on patient-level home blood pressure (HBP).

## Methods

### Patient Population

We retrospectively enrolled patients with chronic cardiovascular diseases (CVDs) who participated in telehealth care at National Taiwan University Hospital (NTUH), Taipei, Taiwan, between January 2009 and December 2013 as the study population. We excluded patients who did not reside in Taipei City during the study period. Informed consent was obtained from all participants. The study was approved by the institutional review board of NTUH. Chronic CVDs included coronary artery disease, prior myocardial infarction, heart failure, peripheral artery disease, prior stroke, and hypertension.

### Telehealth Care Program

In this study, we used a fourth-generation telehealth program developed by Anker et al [13]. The program was designed specifically to offer home care for chronic CVDs. We had previously described the details of this program [14-18]. Briefly, single-lead electrocardiography, BP, heart rate, and oxygen saturation were measured at home, daily and on demand, and the data were instantaneously transmitted to the NTUH Telehealth Center. Case managers would call patients or their caregivers daily and on demand to evaluate patient conditions and provide instructions as needed. There was a call center, and full-time case managers and in-charge cardiologists were available 24 hours a day, 7 days a week. Medical decisions were discussed with patients’ primary care physicians in the case of any major acute event.

### Data Collection

Demographic, clinical (diagnosis of specific diseases), and medication data were obtained from the electronic database of NTUH. All biometric data were measured at home and automatically transmitted to the NTUH server. Biometric data were meant to be measured at least twice daily, ideally after waking up and before sleeping, but each patient or caregiver could have his/her own habit to select a time point and interval within a day to measure biometric parameters. We used the AViTA BPM65ZB sphygmomanometer (AViTA Corp), which is an electronic digital upper arm BP monitor. Hourly meteorological data (including temperature, relative humidity, and wind speed) of Taipei City during the study period were obtained from the Central Weather Bureau, Taiwan. Hourly ambient air pollution monitoring data (including CO, NO_2_, PM_10_, PM_2.5_, and SO_2_) during the same study period were obtained from the Environmental Protection Administration, Taiwan.

### Statistical Analysis

Statistical analysis was performed using the R software (version 3.4.2, The R Foundation for Statistical Computing). In statistical testing, a 2-sided *P* value of ≤.05, was considered significant. The distribution properties of continuous variables are expressed as mean (SD) and median (IQR) values, and categorical variables are presented as frequencies and percentages. The differences in the distributions of continuous variables between male and female subjects were examined using the Wilcoxon rank-sum test. Differences in the distributions of categorical variables were compared using the Fisher exact test. Multivariate analysis was conducted by fitting multiple linear regression models to estimate the adjusted effects of age, sex, comorbidities (including hypertension, diabetes mellitus, cancers, atrial fibrillation, heart failure, prior myocardial infarction, coronary artery disease, prior stroke, and peripheral artery disease), heart rate, antihypertensive agents, seasons, meteorological factors, air pollutants, and other predictors of home systolic and diastolic blood pressure (SBP and DBP).

Since the use of antihypertensive agents, values of meteorological factors, and concentrations of air pollutants varied over time, we defined and included the following three groups of time-dependent covariates in our linear regression analyses:

1. Antihypertensive agents: among the 6 most common classes of antihypertensive drugs, we considered the classes of antihypertensive medications and the number of classes of antihypertensive medications used on the day of BP measurement.
2. Meteorological factors: these included hourly averaged outdoor temperature, relative humidity, and wind speed within the hour of BP measurement.
3. Air pollutants: we determined hourly inverse-distance weighted mean concentrations of 5 air pollutants (CO, NO_2_, PM_10_, PM_2.5_, and SO_2_) and the amount of rainfall within the hour of BP measurement, where the distances were calculated from each patient’s home location to the 6 air quality monitoring stations in Taipei City, based on the corresponding latitudes and longitudes. Instant and cumulated air pollutant concentrations at hours 0, 3, 6, 12, and 18 and days 1, 2, 3, 4, 5, 6, and 7 were included in the multivariate analysis to evaluate the possible lag effect of each pollutant.

Simple and multiple generalized additive models (GAMs) were fitted to assess the nonlinear effects of continuous covariates and identify appropriate cut-off points for discretizing continuous covariates, if necessary, during stepwise variable selection. Further details on statistical analysis are provided in Multimedia Appendix 1.

## Results

### Patient Population and Demographics

A total of 253 patients with CVD who participated in the NTUH Telehealth Care Program from January 2009 to December 2013 were enrolled in this study. A total of 110,715 HBP measurements were carried out accordingly for these patients. The details of this patient population, including the per-patient and per-measurement demographics and clinical characteristics, have already been reported previously [18]. The data distributions of the air pollutant concentrations are summarized in Table 1. The mean temperature, humidity, and wind speed had been reported previously [18]. The mean concentration of NO_2_, PM_10_, PM_2.5_, CO, and SO_2_ were 23.67 (SD 6.80) ppb, 46.83 (SD 21.03) μg/m^3^, 27.96 (SD 10.77) μg/m^3^, 0.74 (SD 0.25) ppm, and 3.05 (SD 1.19) ppb, respectively. The air quality standards recommended by the World Health Organization for these pollutants, during the study period, were based on the 2005 version of the update [19].

**Table 1:** Data distribution of air pollutants from among 110,715 observations obtained by 253 patients included in this study.

Air pollutant	Mean (SD)	Minimum	Maximum	Median
NO_2_ (ppb)	23.67 (6.80)	4.43	63.01	23.19
PM_10_^a^ (μg/m^3^)	46.83 (21.03)	11.64	842.87	42.60
PM_2.5_^b^ (μg/m^3^)	27.96 (10.77)	8.16	140.39	26.07
CO (ppm)	0.74 (0.25)	0.14	2.74	0.70
SO_2_ (ppb)	3.05 (1.19)	0.58	14.85	2.79

^a^PM_10_: particulate matter with a diameter of <10 µm.

^b^PM_2.5_: particulate matter with a diameter of <2.5 µm.

### Multivariate Analysis

**Figure 1:**
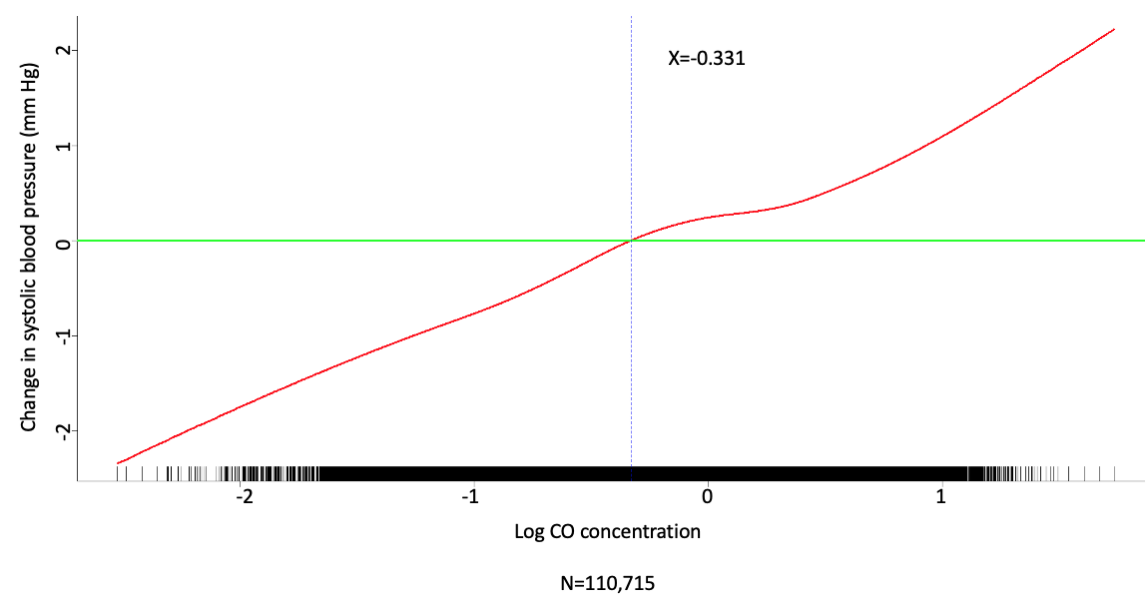
Generalized additive model (GAM) plot showing the relationship between systolic blood pressure and CO exposure.

**Figure 2:**
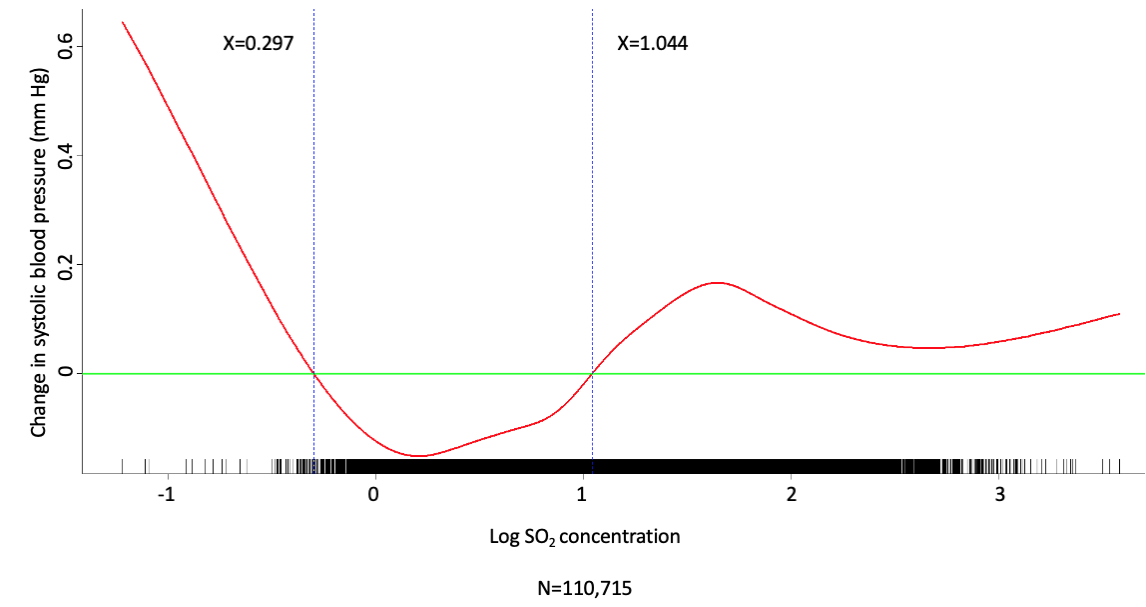
Generalized additive model (GAM) plot showing the relationship between systolic blood pressure and SO_2_ exposure.

**Figure 3:**
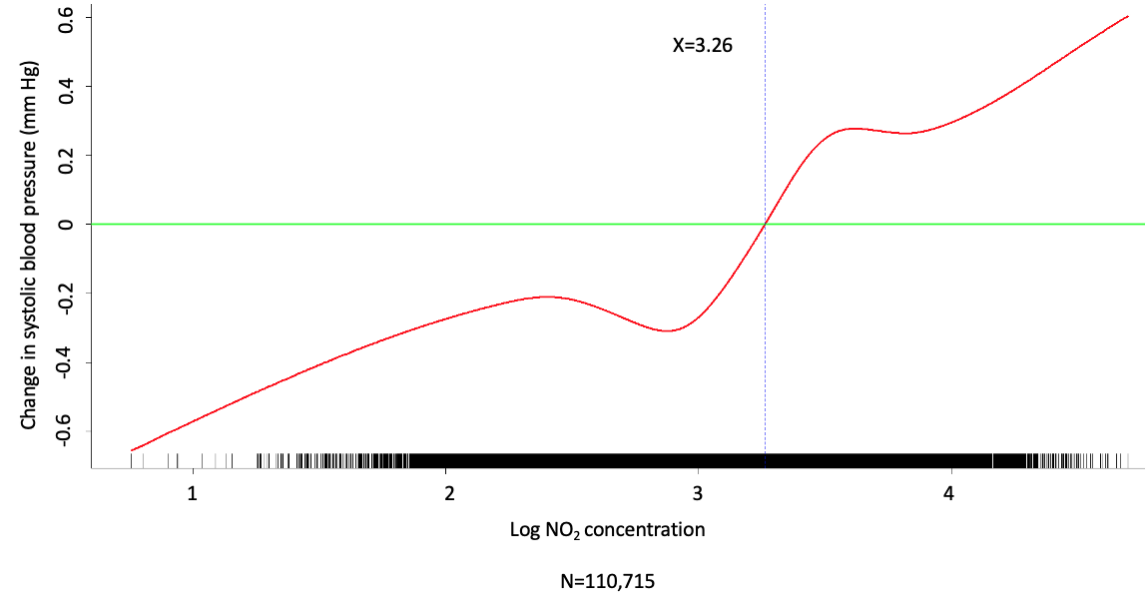
Generalized additive model (GAM) plot showing the relationship between systolic blood pressure and NO_2_ exposure.

**Figure 4:**
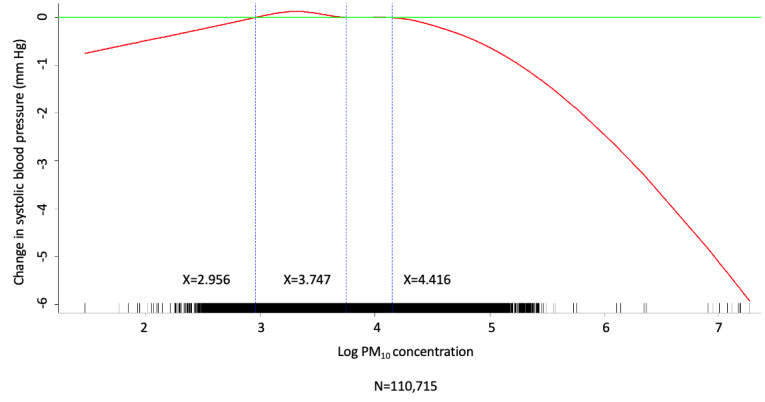
Generalized additive model (GAM) plot showing the relationship between systolic blood pressure and PM_10_ exposure. PM_10_: particulate matter with a diameter of <10 μm.

**Figure 5:**
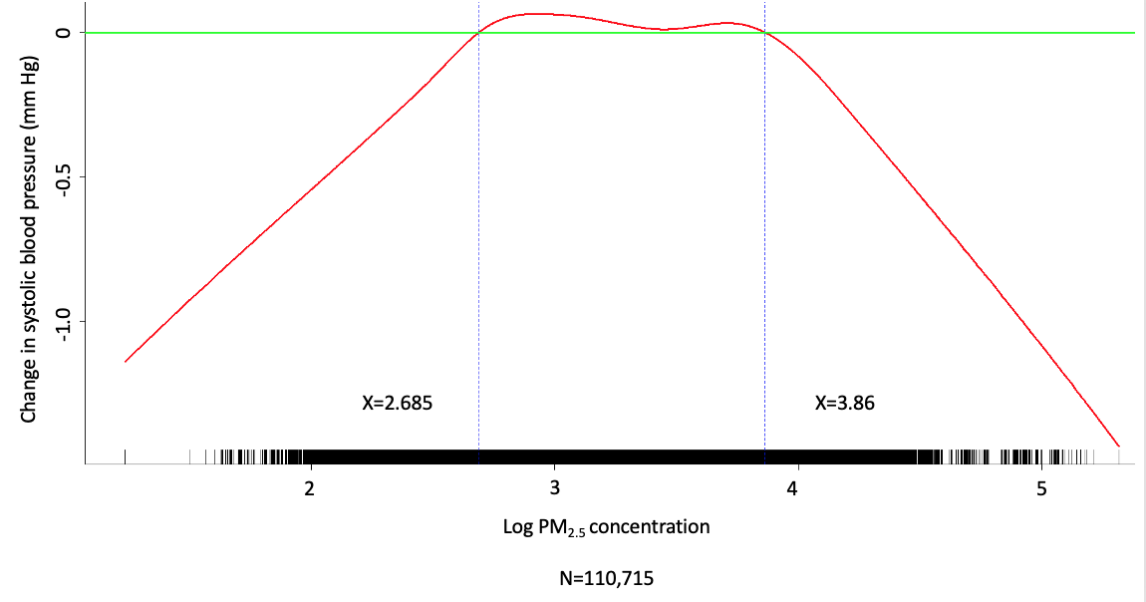
Generalized additive model (GAM) plot showing the relationship between systolic blood pressure and PM_2.5_ exposure. PM_2.5_: particulate matter with a diameter of <2.5 μm.

Multivariate analysis was conducted by fitting multiple linear regression models to estimate the adjusted effects of demographic, clinical, and meteorological factors and air pollutants on home SBP and DBP measurements. The use of antihypertensive agents, values of meteorological factors, and concentrations of air pollutants were all defined and computed as time-dependent covariates. Multivariate analysis of the predictors for SBP and DBP on fitting 1 multiple linear regression model with the stepwise variable selection procedure is shown in Tables 2-7. With regard to SBP and DBP, the significant demographic and clinical characteristics are listed in Tables 2 and 5, meteorological factors in Tables 3 and 6, and air pollutants in Tables 4 and 7, respectively. The hourly averaged temperature had a linear negative effect on both SBP and DBP, while the other meteorological factors had significant nonlinear correlations with home SBP and DBP, as reported previously [18]. The exact statistical data for these meteorological factors were not identical to those reported previously, since this multivariate analysis was performed by using a new model that also included air pollutants. Similarly, CO, NO_2_, SO_2_, PM_2.5_, and PM_10_ concentrations were significantly correlated with home SBP and DBP, and the effects were considered nonlinear. The cut-off points for discretizing the continuous covariates with nonlinear effects on the mean values of SBP and DBP (mmHg) were determined objectively, using the corresponding GAM plots during stepwise variable selection. The GAM plots representing the relationships between SBP and different environmental factors are shown in Figure 1-5. The bar plots of regression coefficient estimates for home SBP and DBP are shown in Multimedia Appendix 2. The green, blue, and red bars represent the demographic and clinical factors, meteorological parameters, and air pollution parameters, respectively. Of note, although the demographic, clinical, meteorological, and air pollution parameters significantly affected home SBP and DBP, it seemed that the “traditional” factors (demographic and clinical) had a more prominent effect on HBP than the environmental factors.

The multiple linear regression model for SBP (n=110,715; *R*^2^=0.1286) indicates that the Pearson correlation coefficient between the observed value of SBP and the model-predicted value of SBP was 0.1286^½^=0.3586, and that for DBP (n=110,715; *R*^2^=0.2219) indicates that the Pearson correlation coefficient between the observed value of DBP and the model-predicted value of DBP was 0.2219^½^=0.4711.

**Table 2:** Multivariate analysis of the predictors for systolic blood pressure by fitting 1 multiple linear regression model with stepwise variable selection: demographic and clinical characteristics.

Covariate	Parameter estimate	SE	*t* value	Pr>|*t*|
Intercept	125.0560	0.8216	152.2027	<.001
Male	–0.5184	0.1133	–4.5771	<.001
AF^a^	0.5663	0.1370	4.1323	<.001
Coronary artery disease without myocardial infarction	3.0135	0.1086	27.7503	<.001
Coronary artery disease with myocardial infarction	–0.5264	0.1708	–3.0820	.002
Cancer	3.0896	0.1402	22.0379	<.001
Chronic heart failure	–4.0580	0.1228	–33.0523	<.001
CVA^b^	1.1025	0.1336	8.2507	<.001
PAOD^c^	–1.7202	0.1855	–9.2744	<.001
ARB^d^ × AB^e^	4.1642	0.4037	10.3161	<.001
ARB × BB^f^	1.8479	0.2601	7.1044	<.001
ARB × CCB^g^	–3.2486	0.2218	–14.6451	<.001
BB	–0.7201	0.1743	–4.1310	<.001
CCB × AB	–8.0115	0.3885	–20.6223	<.001
CCB × ACEI^h^	–8.3039	1.2350	–6.7237	<.001
CCB × BB	–3.9889	0.2950	–13.5204	<.001
CCB × Diuretics	1.0011	0.2187	4.5766	<.001
Diuretics × AB	7.1806	0.2805	25.6013	<.001
Diuretics × ACEI	1.5441	0.4555	3.3902	.001
Diuretics × BB	2.0257	0.2549	7.9458	<.001

^a^AF: atrial fibrillation.

^b^CVA: cardiovascular accident.

^c^PAOD: peripheral arterial occlusion disease.

^d^ARB: angiotensin receptor blocker.

^e^AB: alpha blocker.

^f^BB: beta blocker.

^g^CCB: calcium channel blocker.

^h^ACEI: angiotensin-converting enzyme inhibitor.

**Table 3:** Multivariate analysis of the predictors for systolic blood pressure by fitting 1 multiple linear regression model with stepwise variable selection: meteorological factors.

Covariate	Parameter estimate	SE	*t* value	Pr > |*t*|
Temperature	–0.6352	0.0127	–49.9892	<.001
DM^a^ × temperature	0.1882	0.0045	41.4358	<.001
HTN^b^ × temperature	0.2101	0.0047	44.5223	<.001
ARB^c^ × temperature	0.1528	0.0057	26.6504	<.001
CCB^d^ × temperature	0.0520	0.0078	6.6818	<.001
Diuretics × temperature	–0.0217	0.0059	–3.6496	<.001
0.557 < Wind speed 12 hours ago ≤ 3.73	0.3100	0.1008	3.0752	.002
1.976 < Wind speed on day 0 ≤ 4.43	0.5216	0.1099	4.7471	<.001
1.983 < Wind speed 1 day ago ≤ 3.895	0.2267	0.1044	2.1707	.03
1.793 < Wind speed 2 days ago ≤ 3.634	0.3138	0.0990	3.1702	.002
1.587 < Wind speed 4 days ago ≤ 3.923	0.2128	0.0995	2.1380	.03
1.855 < Wind speed 6 days ago ≤ 3.575	0.3155	0.0979	3.2221	.001
Relative humidity ≤ 65.774 or > 84.596	0.5365	0.1000	5.3641	<.001
Relative humidity 6 hours ago ≤ 72.967 or > 92.905	0.4753	0.1096	4.3364	<.001
Relative humidity 12 hours ago ≤ 56.324 or > 78.989	0.3813	0.1178	3.2365	.001
Relative humidity 24 hours ago > 76.11	0.4278	0.1209	3.5391	<.001
Relative humidity 2 days ago ≤ 67.752 or > 82.532	0.3446	0.1003	3.4362	.001
Relative humidity 4 days ago ≤ 65.366 or > 82.318	0.4172	0.1044	3.9960	<.001
Relative humidity 6 days ago ≤ 58.849 or > 81.108	0.5261	0.1331	3.9527	<.001
Log rainfall	–0.2074	0.0593	–3.4984	.001
Log cumulated rainfall in the past 4 days	–0.2981	0.0684	–4.3564	<.001
Log rainfall 6 days ago < –0.106	0.6989	0.1652	4.2296	<.001

^a^DM: diabetes mellitus.

^b^HTN: hypertension.

^c^ARB: angiotensin receptor blocker.

^d^CCB: calcium channel blocker.

**Table 4:** Multivariate analysis of the predictors for systolic blood pressure by fitting 1 multiple linear regression model with stepwise variable selection: air pollutants.

Covariate	Parameter estimate	SE	*t* value	Pr>|*t*|
Log CO concentration 3 hours ago	–0.6620	0.1186	–5.5800	<.001
Log cumulated CO concentration in the past 5 days	1.1985	0.2965	4.0422	<.001
Log NO_2_ concentration	0.4520	0.1493	3.0276	.003
2.571 < Log NO_2_ concentration 3 hours ago ≤ 3.654	0.3311	0.1113	2.9752	.003
Log NO_2_ concentration 6 hours ago	0.7517	0.1154	6.5136	<.001
2.188 < Log NO_2_ concentration 18 hours ago ≤ 3.185	0.4333	0.0986	4.3971	<.001
3.062 < Log NO_2_ concentration 3 days ago ≤ 3.497	0.4392	0.1026	4.2810	<.001
3.063 < Log NO_2_ concentration 4 days ago ≤ 3.483	0.3689	0.1027	3.5929	<.001
2.567 < Log NO_2_ concentration 5 days ago ≤ 3.507	0.4131	0.1435	2.8781	.004
3.059 < Log NO_2_ concentration 6 days ago ≤ 3.494	0.4837	0.1037	4.6650	<.001
3.043 < Log NO_2_ concentration 7 days ago ≤ 3.468	0.3438	0.1004	3.4256	.001
Log PM_2.5_^a^ concentration 3 hours ago < 2.284	1.1068	0.3245	3.4105	.001
Log PM_2.5_ concentration 4 days ago ≤ 3.243 or > 4.378	0.5000	0.1031	4.8481	<.001
Log PM_2.5_ concentration 6 days ago ≤ 3.034 or > 3.826	0.3055	0.1076	2.8383	.005
2.848 < Log PM_10_^b^ concentration 18 hours ago ≤ 3.828	0.4819	0.1032	4.6690	<.001
Log SO_2_ concentration 3 hours ago < –0.271	3.5314	1.0028	3.5216	<.001
Log SO_2_ concentration 24 hours ago > 1.561	0.4552	0.1372	3.3164	.001
Log SO_2_ concentration 5 days ago > 1.316	0.2970	0.1187	2.5025	.01

^a^PM_2.5_: particulate matter with a diameter of <2.5 µm.

^b^PM_10_: particulate matter with a diameter of <10 µm.

**Table 5:** Multivariate analysis of the predictors for diastolic blood pressure by fitting 1 multiple linear regression model with stepwise variable selection: demographic and clinical characteristics.

Covariate	Parameter estimate	SE	*t* value	Pr>|*t*|
Intercept	65.7236	0.7686	85.5088	<.001
Male	–1.2416	0.0793	–15.6608	<.001
Age < 72.424 years	8.5878	0.0783	109.6523	<.001
AF^a^	–0.8389	0.0916	–9.1609	<.001
Coronary artery disease with myocardial infarction	–0.3166	0.1088	–2.9105	.004
Cancer	0.4197	0.0942	4.4572	<.001
CHF^b^	–0.8912	0.0806	–11.0540	<.001
CVA^c^	3.2332	0.0896	36.0651	<.001
PAOD^d^	1.8971	0.1279	14.8373	<.001
ARB^e^ × AB^f^	6.8305	0.2722	25.0906	<.001
ARB × BB^g^	5.6929	0.1636	34.7953	<.001
ARB × CCB^h^	–3.3888	0.1369	–24.7458	<.001
ARB × diuretics	–3.4067	0.1401	–24.3191	<.001
CCB × ACEI^i^	–10.7720	0.8297	–12.9827	<.001
CCB × BB	–0.4592	0.1868	–2.4583	.01
CCB × diuretics	2.2884	0.1298	17.6274	<.001
Diuretics × AB	–3.4526	0.2494	–13.8457	<.001
Diuretics × ACEI	2.5617	0.3943	6.4973	<.001
Diuretics × BB	–1.7401	0.1616	–10.7675	<.001

^a^AF: atrial fibrillation.

^b^CHF: congestive heart failure.

^c^CVA: cerebrovascular accident.

^d^PAOD: peripheral occlusive arterial disease.

^e^ARB: angiotensin receptor blocker.

^f^AB: alpha blocker.

^g^BB: beta blocker.

^h^CCB: calcium channel blocker.

^i^ACEI: angiotensin converting enzyme inhibitor.

**Table 6:** Multivariate analysis of the predictors for diastolic blood pressure by fitting 1 multiple linear regression model with stepwise variable selection: meteorological factors.

Covariate	Parameter estimate	SE	*t* value	Pr>|*t*|
Temperature	–0.1608	0.0130	–12.3600	<.001
Temperature 12 hours ago	–0.1058	0.0135	–7.8265	<.001
Temperature 18 hours ago ≤ 16.992 or > 28.649	0.5042	0.0714	7.0590	<.001
Temperature 7 days ago	0.0753	0.0124	6.0722	<.001
DM^a^ × temperature	0.0694	0.0030	22.9718	<.001
HTN^b^ × temperature	0.0857	0.0032	26.9498	<.001
AB^c^ × temperature	–0.1161	0.0083	–13.9588	<.001
ACEI^d^ × temperature	–0.0323	0.0096	–3.3598	.001
ARB^e^ × temperature	0.0623	0.0046	13.5546	<.001
Diuretics × temperature	–0.0177	0.0045	–3.9649	<.001
Wind speed	0.1122	0.0250	4.4919	<.001
Wind speed 18 hours ago	0.0612	0.0230	2.6618	.008
Wind speed 3 days ago	0.1358	0.0318	4.2695	<.001
Wind speed 5 days ago > 3.734	0.3835	0.0892	4.3014	<.001
Wind speed 7 days ago > 4.56	0.7900	0.1750	4.5145	<.001
Relative humidity 6 hours ago ≤ 67.108 or > 91.436	0.3259	0.0719	4.5359	<.001
Relative humidity 12 hours ago ≤ 57.1 or > 78.822	0.2567	0.0836	3.0700	.002
Relative humidity 18 hours ≤ 34.851 or >77.249	0.2478	0.0983	2.5216	.01
Relative humidity 24 hours ago ≤ 47.631 or >77.286	0.2813	0.0893	3.1501	.002
Relative humidity 2 days ago ≤ 64.191 or > 81.286	0.2489	0.0728	3.4188	.001
Relative humidity 4 days ago ≤ 64.291 or > 81.392	0.3521	0.0729	4.8281	<.001
Relative humidity 5 days ago ≤ 54.611 or > 76.3	0.3089	0.0852	3.6268	<.001
Relative humidity 6 days ago ≤ 54.383 or > 79.265	0.5044	0.0985	5.1202	<.001
Relative humidity 7 days ago ≤ 58.455 or >76.46	0.5243	0.0856	6.1239	<.001
Log rainfall > –2.252	0.3004	0.0824	3.6452	<.001
Log rainfall 3 hours ago ≤ –0.372 or > 0.817	0.4580	0.1538	2.9783	.003
–2.251 < Log rainfall 6 hours ago ≤ 0.379	0.3796	0.0856	4.4340	<.001
–2.246 < Log rainfall 12 hours ago ≤ 0.177	0.3282	0.0874	3.7542	<.001
–2.228 < Log rainfall 18 hours ago ≤ 3.202	0.1674	0.0846	1.9797	.05
–2.165 < Log rainfall 2 days ago ≤ 0.145	0.4425	0.0681	6.4961	<.001
–2.168 < Log rainfall 4 days ago ≤ 0.068	0.3491	0.0682	5.1162	<.001
–2.153 < Log rainfall 5 days ago ≤ 0.026	0.3042	0.0691	4.4003	<.001
–2.193 < Log rainfall 7 days ago ≤ –0.266	0.5065	0.0666	7.6091	<.001
–2.044 < Log cumulated rainfall in the past 6 days ≤ –0.735	0.5517	0.0691	7.9805	<.001
Log cumulated rainfall in the past 7 days	–0.5756	0.0538	10.7067	<.001

^a^DM: diabetes mellitus.

^b^HTN: hypertension.

^c^AB: alpha blocker.

^d^ACEI: angiotensin converting enzyme inhibitor.

**Table 7:** Multivariate analysis of the predictors for diastolic blood pressure by fitting 1 multiple linear regression model with stepwise variable selection: air pollutants.

Covariate	Parameter estimate	SE	*t* value	Pr>|*t*|
Log CO concentration	0.3966	0.0970	4.0907	<.001
Log CO concentration 12 hours ago	–0.9318	0.0759	–12.2815	<.001
–0.41 < Log CO concentration 24 hours ago ≤ 0.527	0.4100	0.0713	5.7517	<.001
Log CO concentration 2 days ago ≤ –0.426 or > 0.366	0.2789	0.0743	3.7546	<.001
Log cumulated CO concentration in the past 5 days > 0.034	1.0558	0.2096	5.0366	<.001
Log cumulated CO concentration in the past 7 days > 0.006	0.7773	0.2050	3.7921	<.001
Log NO_2_ concentration 6 hours ago	0.1645	0.0763	2.1566	.03
2.226 < Log NO_2_ concentration 18 hours ago ≤ 3.209	0.3400	0.0689	4.9383	<.001
Log cumulated NO_2_ cconcentration in the past 1 day > 3.163	0.1880	0.0887	2.1203	.03
Log NO_2_ concentration 2 days ago < 2.695	0.6081	0.1352	4.4984	<.001
3.1 < Log NO_2_ concentration 3 days ago ≤ 3.6	0.2480	0.0727	3.4093	.007
Log NO_2_ concentration 7 days ago	–0.9739	0.1572	–6.1965	<.001
Log PM_2.5_^a^ concentration 1 day ago ≥ 2.734	0.6872	0.1397	4.9188	<.001
Log PM_2.5_ concentration 2 days ago > 3.547	0.2638	0.0871	3.0294	.003
Log PM_2.5_ concentration 3 days ago ≥ 2.598	0.4660	0.1869	2.4936	.01
Log PM_2.5_ concentration 6 days ago > 3.559	0.2502	0.0827	3.0256	.003
Log PM_10_^b^ concentration 1 day ago	–0.5895	0.1264	–4.6622	<.001
0.926 < Log SO_2_ concentration ≤ 2.325	0.2053	0.0768	2.6736	.008
0.77 < Log SO_2_ concentration 3 hours ago ≤ 1.902	0.1994	0.0718	2.7766	.006
0.73 < Log SO_2_ concentration 18 hours ago ≤ 1.819	0.2264	0.0683	3.3144	.001
1.062 < Log SO_2_ concentration 24 hours ago ≤ 2.926	0.2025	0.0726	2.7908	.005
0.772 < Log SO_2_ concentration 5 days ago ≤ 1.558	0.2316	0.0696	3.3270	.001
0.946 < Log SO_2_ concentration 6 days ago ≤ 1.604	0.2500	0.0682	3.6660	<.001
Log SO_2_ concentration 7 days ago	0.5296	0.1162	4.5578	<.001

^a^PM_2.5_: particulate matter with a diameter of <2.5 µm.

^b^PM_10_: particulate matter with a diameter of <10 µm.

## Discussion

### Principal Findings

Human health is significantly correlated with genomic and environmental factors. Wild [20] defined the “exposome” in 2005 as a concept that “encompasses life-course environmental exposures (including lifestyle factors), from the prenatal period onwards.” Among these factors in the exposome, air pollution poses a major threat to human health. Exposure to ambient air pollution, either over the short or long term, increases cardiovascular mortality accordingly [6,8]. The detrimental effect of air pollution on BP is considered one of the mechanisms leading to this outcome. However, the association between short-term or long-term exposure to ambient air pollution and BP is inconsistent in the literature. Among the routinely monitored air pollutants, PM_2.5_ is considered one of the most dangerous agents influencing human health. Brook et al [8] adequately described the association between particulate matter air pollution and CVD, suggesting that exposure to ambient PM_2.5_ would potentially increase BP. A few mechanisms explaining why PM_2.5_ increases BP have been reported before: (1) altering the autonomic nervous system to favor a sympathetic tone [21], (2) increasing oxidative stress through endogenous proinflammatory mediators or vasculo-active molecules [22,23], (3) directly influencing the vascular endothelium [24,25], and (4) inducing abnormal DNA methylation [26,27]. Many studies have investigated the association between exposure to ambient PM_2.5_ and BP, which has been reported before, but the effect was inconsistent. To date, 4 meta-analyses have addressed this issue. Liang et al [9] reported positive associations among PM_2.5_, SBP, and DBP. Zhang et al [10] reported that a high SBP is significantly associated with the exposure to PM_2.5_, while a high DBP is only associated with the exposure to PM_10_, but not PM_2.5_. Cai et al [11] reported that short-term exposure to SO_2_, PM_2.5_, and PM_10_ was significantly associated with an increased risk of hypertension. The association between BP and air pollutants other than PM has not been investigated in detail. In a recent meta-analysis by Yang et al [12], short-term exposure to ambient air pollution was significantly associated with hypertension (PM_10_, PM_2.5_, SO_2_, and NO_2_), SBP (PM_2.5_ and SO_2_), and DBP (PM_10_, PM_2.5_, SO_2_, and NO_2_). However, these meta-analyses were mainly performed on the basis of epidemiological studies. To our knowledge, our study is the first to investigate the relationships among demographic, clinical, meteorological factors, air pollutants, and HBP at the patient level.

There are some notable findings in this study. First, at the patient level, ambient air pollution was significantly associated with HBP, both SBP and DBP, and the culprit pollutants included all of the 5 pollutants (PM_10_, PM_2.5_, SO_2_, NO_2_, and CO) that were included in this study. The association between HBP and ambient air pollution was surprisingly nonlinear, given that most previous studies used a linear model to evaluate the effect of ambient air pollution on BP and hypertension. Yang et al [12] suspected that the relationship between ambient air pollution and BP was nonlinear, and this may be the reason why even meta-analyses that have been well performed could not adequately explain the association between ambient air pollution and BP because most previous studies mistakenly used a linear model. One of the strengths of this study was that we considered HBP rather than office BP measurements, which was the case in most of the previous studies. HBP is now considered a more important predictor than office BP for future coronary and cerebral events [28]. All ambient air pollutants investigated in this study significantly affected HBP, while in prior meta-analyses, only some of the ambient air pollutants were significantly associated with hypertension, SBP, and DBP. This could explain why ambient air pollution poses a great hazard to human health. Data regarding medication in this study are also important. No study thus far has investigated whether antihypertensive agents play a role in the association between ambient air pollution and BP. Our prior study [18] showed that short-term exposure to low ambient temperature significantly increases HBP, and that this effect could be modified by antihypertensive agents. In this study, the potential confounding effect of medications was also considered, but ambient air pollutants still showed significant associations with HBP. Limited by the statistical model, the exact effect of each class of antihypertensive agents on the relationship between ambient air pollution and HBP could not be fully elucidated. Of note, although there were significant associations between environmental factors and HBP, demographic and clinical factors seemed to play an even more important role in HBP, as shown in Multimedia Appendix 2. Nonetheless, since hypertension has such a marked impact on human health, a holistic approach for BP control, including adequate modification of environmental exposure (both meteorological factors and ambient air pollutants), should be adopted. The importance of air pollution control cannot be overemphasized.

### Limitations

This study has a few limitations. First, this was a retrospective registry with a relatively small number of patients. Second, the study population comprised patients with chronic CVD with great adherence and health insights as they opted to participate in a telehealth care program. Thus, whether the study results can be extrapolated to other patient populations should be carefully considered. Third, individual ambient air pollutant concentrations were calculated using a spatial model that might not reflect true personal exposure. Jiang et al [29] reported that even people who were geographically close could have distinct personalized exposomes. Without precise assessment of personal environmental exposure, further evaluation of the associations between the environment and human health seems futile. Jiang et al [29] reported that novel wearable devices that are capable of assessing both biotic and abiotic exposure may be used to solve this problem. As shown in our previous study [18] and in this study, meteorological factors (including temperature, relative humidity, and wind speed) and ambient air pollutants (NO_2_, PM_10_, PM_2.5_, CO, and SO_2_) had significant effects on human health at the patient level and should be considered in future studies on abiotic exposure among exposomes. Fourth, the possible interactions among air pollutants, including synergistic effects and collinearity, were not addressed in this study. Fifth, this study reports a nonlinear relationship between BP and ambient air pollutants, but further evaluation of the dose-effect curve was limited by the current statistical methods. Similarly, the interactions between ambient air pollution and other environmental factors, such as meteorological factors, were not addressed in this study. Multiscale entropy is a novel nonlinear method that has been applied to predict outcomes in a few clinical settings, including trauma [30], after cardiac arrest on mechanical circulatory support [31], acute stroke [32], and autonomic imbalance after myocardial infarction [33]. Further studies may also apply this method to evaluate the effect of ambient air pollution on BP. Sixth, this study only investigated the effect of short-term exposure to ambient air pollution; the effect of long-term exposure remains unknown. Seventh, ozone is an important air pollutant associated with an elevated BP [34-38]. However, during the study period (2009-2013), ozone was not routinely monitored by the Environmental Protection Administration in Taiwan; hence, it was not feasible to evaluate the relationship between ozone and HBP in the current study. Finally, the lag effect of each air pollutant on BP was not precisely determined in this study because of the complexity of the statistical analysis.

### Conclusions

Short-term exposure to ambient air pollution significantly affects both home SBP and DBP in patients with chronic CVD, and the relationship between ambient air pollution and HBP is nonlinear.

## Appendix

Multimedia Appendix 1 More Statistical Details.

Multimedia Appendix 2 Additional figure and figure legends.

## Abbreviations

BP: blood pressure
CVD: cardiovascular disease
DBP: diastolic blood pressure
GAM: generalized additive models
HBP: home blood pressure
NTUH: National Taiwan University Hospital
PM_10_: particulate matter with a diameter of <10 μm
PM_2.5_: particulate matter with a diameter of <2.5 μm
SBP: systolic blood pressure
