# Supplementary material for: The Association Between Short-term Exposure to Ambient Air Pollution and Patient-Level Home Blood Pressure Among Patients With Chronic Cardiovascular Diseases in a Web-Based Synchronous Telehealth Care Program: Retrospective Study
Source: JMIR Public Health Surveill. 2021 Jun 8;7(6):e26605. doi: 10.2196/26605 (PMC8238492; doi:10.2196/26605)
Supplement: Multimedia Appendix 1 [file publichealth_v7i6e26605_app1.docx]

**Multimedia Appendix 1**

**More Statistical Details.**

**Univariate and multivariate analyses.**

The goal of regression analysis was to find one or a few parsimonious regression models that fitted the observed data well for effect estimation and/or outcome prediction. To ensure a good quality of analysis, the model-fitting techniques for (1) variable selection, (2) goodness-of-fit (GOF) assessment, and (3) regression diagnostics and remedies were used in our linear regression analyses. Specifically, the stepwise variable selection procedure (with iterations between the forward and backward steps) was applied to obtain the best candidate final linear regression model using the My.stepwise.lm() function of the My.stepwise package in R (Hu, 2017). All the univariate significant and non-significant relevant covariates (listed in Table 1) and some of their interaction terms (or moderators) were put on the variable list to be selected. The significance levels for entry (SLE) and for stay (SLS) were set to 0.15 for being conservative. Then, with the aid of substantive knowledge, the best candidate final linear regression model was identified manually by dropping the covariates with p value > 0.05 one at a time until all regression coefficients were significantly different from 0. Any discrepancy between the results of univariate analysis and multivariate analysis was likely due to the confounding effects of uncontrolled covariates in univariate analysis or the masking effects of intermediate variables (or mediators) in multivariate analysis.

The GOF measure, coefficient of determination R , was examined to assess the

GOF of the fitted linear regression model. Technically, the R2 statistic (0 ≤ R2 ≤ 1) for linear regression model equals the square of the Pearson correlation between the observed and predicted response values and it indicates how much of the response variability is explained by the covariates included in the linear regression model.

**Stepwise variable selection.** Computationally, the vgam() function (with the default values of smoothing parameters) of the VGAM package (Yee and Wild, 1996; Yee, 2015, 2017) was used to fit GAMs for our continuous responses in R. Finally, the statistical tools of regression diagnostics for residual analysis, detection of influential cases, and check of multicollinearity were applied to discover any model or data problems. The values of variance inflating factor (VIF) ≥ 10 in continuous covariates or ≥ 2.5 in categorical covariates indicate the occurrence of the multicollinearity problem among some of the covariates in the fitted linear regression model.
