# Supplementary material for: The Association Between Short-term Exposure to Ambient Air Pollution and Patient-Level Home Blood Pressure Among Patients With Chronic Cardiovascular Diseases in a Web-Based Synchronous Telehealth Care Program: Retrospective Study
Source: JMIR Public Health Surveill. 2021 Jun 8;7(6):e26605. doi: 10.2196/26605 (PMC8238492; doi:10.2196/26605)

**Multimedia Appendix 2**

**ADDITIONAL FIGURE LEGENDS**

Additional Figure 1. The bar plot of regression coefficient estimates for SBP. The green, blue and red bars represented for demographic/clinical, meteorological and air pollution parameters, respectively. It is the same in the Additional figure 2.

Additional Figure 2. The bar plot of regression coefficient estimates for DBP.


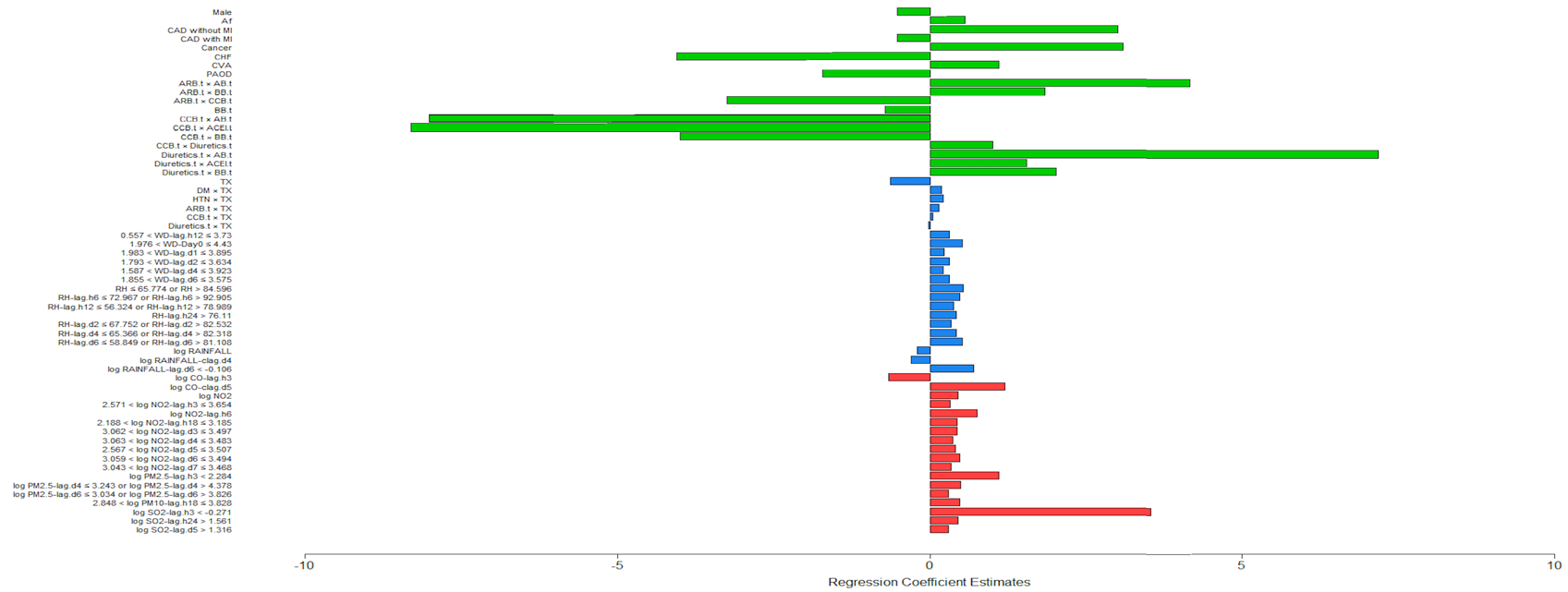
**Additional Figure 1**

**Additional Figure 2**


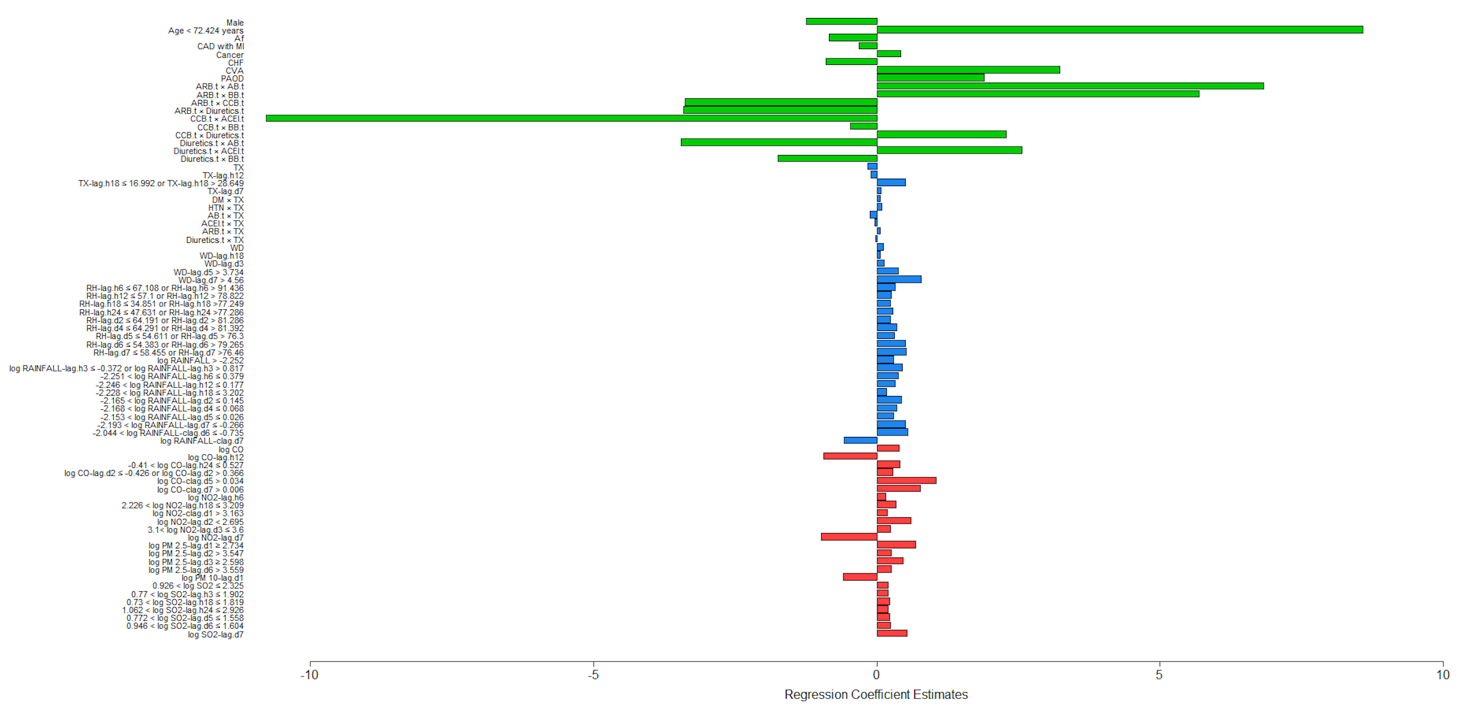

Supplement: Multimedia Appendix 2 [file publichealth_v7i6e26605_app2.docx]
